# Enhancer–promoter specificity in gene transcription: molecular mechanisms and disease associations

**DOI:** 10.1038/s12276-024-01233-y

**Published:** 2024-04-25

**Authors:** Meyer J. Friedman, Tobias Wagner, Haram Lee, Michael G. Rosenfeld, Soohwan Oh

**Affiliations:** 1 Department and School of Medicine, University of California, San Diego, La Jolla, CA USA; 2 College of Pharmacy Korea University, 2511 Sejong-ro, Sejong, 30019 Republic of Korea

**Keywords:** Transcriptional regulatory elements, Chromatin structure

## Abstract

Although often located at a distance from their target gene promoters, enhancers are the primary genomic determinants of temporal and spatial transcriptional specificity in metazoans. Since the discovery of the first enhancer element in simian virus 40, there has been substantial interest in unraveling the mechanism(s) by which enhancers communicate with their partner promoters to ensure proper gene expression. These research efforts have benefited considerably from the application of increasingly sophisticated sequencing- and imaging-based approaches in conjunction with innovative (epi)genome-editing technologies; however, despite various proposed models, the principles of enhancer–promoter interaction have still not been fully elucidated. In this review, we provide an overview of recent progress in the eukaryotic gene transcription field pertaining to enhancer–promoter specificity. A better understanding of the mechanistic basis of lineage- and context-dependent enhancer–promoter engagement, along with the continued identification of functional enhancers, will provide key insights into the spatiotemporal control of gene expression that can reveal therapeutic opportunities for a range of enhancer-related diseases.

## Enhancers in focus: insights into spatiotemporal gene expression control

This review summarizes the current understanding of enhancer-promoter interactions (EPIs), a key feature of genome structure, in gene regulation and disease. EPIs are crucial for proper gene activity, and their alteration by mutational events can be pathological, causing, among others, cancer, and neurodevelopmental disorders. The authors provide an overview of various methods used to study 3D genome architecture (the physical structure of chromosomes) and discuss recent insights that these techniques have afforded, especially with regard to the specificity and dynamics of EPIs as well as the underlying molecular mechanisms. The review highlights the broad experimental utility of CRISPR-based strategies and their therapeutic potential for targeting disease-related EPIs. While substantial progress has been made, further elucidation of the physical and functional interplay of enhancers with their cognate promoter(s) will reveal new treatment opportunities for many diseases.

This summary was initially drafted using artificial intelligence, then revised and fact-checked by the author.

## Introduction

Transcriptional regulation is a crucial feature of gene expression control during development, homeostasis, and signal-dependent responses. In eukaryotes, DNA sequences located proximal or distal to the transcription start site (TSS), referred to as *cis*-regulatory elements (CREs), play pivotal roles in the regulation of RNA polymerase II (Pol II)-dependent gene expression. The CRE that is closest to the TSS (typically <1 kb) is the promoter, which, when active, recruits transcriptional machinery and is marked by trimethylation of histone H3 on lysine 4 (H3K4me3). More remotely positioned CREs (>1 kb from the TSS) serve as enhancers, the active versions of which also feature open or accessible chromatin occupied by transcriptional complexes. Active enhancers often display higher levels of H3K4 monomethylation (H3K4me1) than promoters, and they tend to show differential engagement of lineage-determining and cell type-specific transcription factors (TFs), based on the presence of specific DNA motifs, in addition to certain transcriptional coactivators, such as p300 and MED1^1^. Despite these differences, functional enhancers and promoters share some common characteristics, including nucleosome-free DNase hypersensitivity, enrichment of H3K27ac as well as other active histone marks, recruitment of general and specific TFs, and bidirectional transcription. Indeed, it is now apparent that the classification of promoters and enhancers, at least from a functional perspective, can be arbitrary^2^. Nevertheless, a comprehensive understanding of enhancer and promoter grammar and functionality, including their interplay, is necessary to elucidate gene regulatory strategies in normal physiology and potential pathological alterations.

A number of large-scale research projects have sought to catalog and characterize CREs. Notably, the ENCODE consortium has interrogated nearly a million putative CREs in the human genome by examining multiple epigenomic features^3^. Recent advances in single-cell technologies have allowed for the analysis of cell type-specific CREs. While application of single-cell ATAC-seq has proven particularly powerful for annotation of putative CREs, additional single-cell-based strategies have begun to provide more extensive epigenomic profiling of CREs in distinct cell types from various tissues during different developmental stages or in certain disease contexts, even though technical challenges regarding cell number and quality as well as in data processing remain^4,5^. In addition, high-throughput, sequencing-based reporter assays, such as self-transcribing active regulatory region sequencing (STARR-seq) and massively parallel reporter assays (MPRAs), have provided complementary insights into the functional potential and properties of natural as well as artificial CREs^6^.

Validation of putative CREs requires assignment of their target genes, which is critical for discerning any role in transcriptional regulation. Although this process is typically straightforward for promoter CREs, given their location at the TSS(s) of annotated genes, it is a considerably more challenging task for distal CREs that constitute enhancers. High-throughput derivatives of chromatin conformation capture (3 C), which detect physical contacts between genomic regions, and approaches based on expression quantitative trait loci (eQTL) that correlate genetic variants to gene transcript levels have helped in identifying instances of enhancer–promoter interaction (EPI), which is generally considered to be a key feature of enhancer-mediated regulation^7,8^. Nevertheless, a better understanding of the molecular mechanisms by which enhancers interact with their target genes to activate transcription is required to elucidate regulatory strategies. In this review, we provide an overview of recent insights into the molecular underpinnings of EPI in the context of 3D genome architecture and discuss the disease implications of EPI alterations. In addition, we briefly introduce the experimental methods used to uncover EPIs and comment on issues with data interpretation as well as discrepancies arising from different approaches.

### Identification of enhancer-promoter interactions

It is now apparent that eukaryotic genomes are highly organized within the nucleus and that the prevailing 3D structure, which is relatively stable but amenable to alterations, impacts transcription and ultimately cellular phenotype. Fundamental features of genomic organization, including topologically associated domains (TADs) and the polymeric nature of chromatin, have been elucidated by utilization of 3C-based techniques combined with next-generation sequencing (NGS) and super-resolution microscopy tools^9,10^. EPIs occur within the context of hierarchical 3D genomic architecture. Although functional validation of most EPIs is lacking, the regulatory role of enhancer–promoter looping has been confirmed in many studies of individual loci and can be addressed in a high-throughput manner with clustered regularly interspaced short palindromic repeats (CRISPR)-based epigenome editing approaches.

### 3C-based assays and complementary approaches

**Fig. 1:**
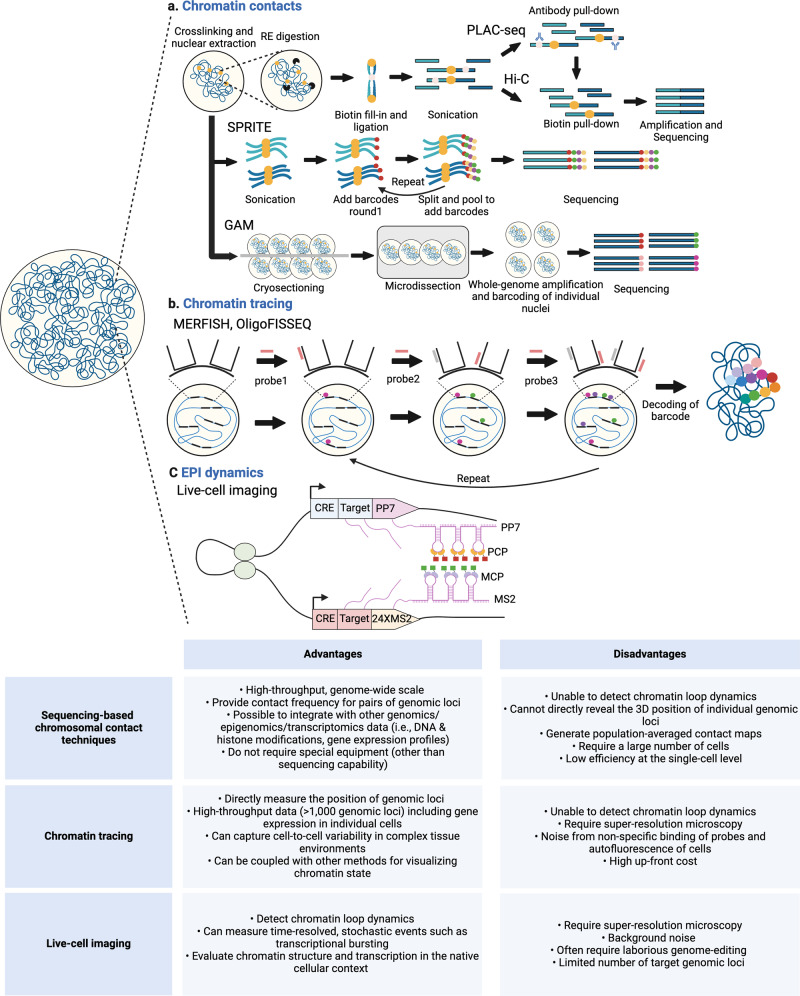
Schematic representation of methods for interrogation of EPIs and 3D genome organization. 3D genome architectural features, including EPIs, have been studied in bulk or in single cells by combining methods that probe DNA‒DNA, DNA‒RNA, and DNA‒protein interactions with sequencing and microscopy approaches. **a** Hi-C, a genome-wide 3C-based technique, and its derivatives have profoundly impacted investigation of the 3D genome. Contacts between genomic loci can be captured by Hi-C at kilobase-scale resolution, but this method requires massive sequencing reads. To achieve high resolution with lower sequencing costs, protein-centric immunoprecipitation was combined with Hi-C, yielding PLAC-seq, which is also known as HiChIP. Ligation-free methods, such as SPRITE and GAM, were developed to minimize artifacts arising from proximity ligation. **b** Multiplexed oligo-based FISH allows for systematic direct tracing of 3D genome structure and has been adapted to visualize promoters and enhancers by targeting histone modifications. **c** Actively transcribing genomic loci, such as genes and enhancers, can be monitored simultaneously with two-color, live-cell imaging by visualizing genomically integrated MS2/PP7 repeats via fluorescently tagged-versions of their respective RNA-binding proteins, MCP and PCP.

A large collection of methods has been introduced to enable the study of chromosome architecture that broadly includes 3C and its derivatives, which involve proximity ligation of digested chromosomes in crosslinked cells, as well as alternative procedures with sequencing readouts that do not rely on a ligation step, namely split-pool recognition of interactions by tag extension (SPRITE) and genome architecture mapping (GAM) (Fig. 1a)^11^. 3C-based techniques include 4C-seq and Hi-C, which has various derivatives itself, such as PLAC-seq, Capture-C, and micro-C^11^, and also has been optimized^12^. A crosslinking-independent, 3C-based assay, known as intrinsic 3C, has also been developed^13^. Collectively, these 3C-based techniques have revealed the presence of territories in metazoan chromosomes that are further organized into A/B compartments, TADs, and chromatin loops that include EPIs^14^. Despite substantial advancements in the understanding of 3D genome architecture afforded by 3C-based assays, proximity ligation methods have inherent limitations. First, in instances that involve contacts connecting three or more genomic loci, these methods cannot clearly distinguish whether the interaction is simultaneous within the same cell or occurs in a pairwise manner in different cells. Second, these strategies are not able to detect long-range DNA interactions that cannot be efficiently ligated^15^. To overcome these challenges, several modified procedures, such as C-walks (molecular barcoding), MC-4C (long-read sequencing), and Pore-C (nanopore sequencing), attempt to capture both pairwise and multiway chromosomal contacts by sequencing large proximity-ligated concatemers to reveal high-order conformations. Recently developed genome-wide, ligation-free approaches, including SPRITE, GAM, and ChIA-Drop, can also survey multiway chromosomal contacts and are not affected by artifacts arising from proximity ligation; however, these assays still require crosslinking. SPRITE involves sequencing barcoded DNA following multiple rounds of split-pool tagging, with the expectation that each interacting chromatin complex will have a distinct barcode. Thus, an interaction map can be determined by compiling the retrieved DNA segments with the same barcode. In the GAM technique, interaction data are gathered from thin cryosections of fixed cell nuclei that are collected by laser microdissection. Contact frequencies can be inferred because adjacent DNA loci are more likely to be present in the same nuclear slice. In ChIA-Drop, a chromatin complex isolated by ChIP is subjected to droplet-based sequencing. As the contents of each droplet are uniquely barcoded, the interaction map can be determined in a manner similar to that of SPRITE but with single-molecule resolution. Notably, several years after their respective development, SPRITE and GAM have yet to be widely adopted, possibly due to distinct technical challenges associated with implementation of each technique.

### Imaging-based results

In parallel, application of quantitative imaging methods has revealed molecular features of EPI in single cells. Aspects of genome structure, including the spatial distance between two or more chromosomal loci, can be directly visualized in both fixed and live cells by high-resolution microscopy.

Advancements in DNA/RNA detection and microscopy techniques have facilitated imaging of EPI. While standard fluorescence in situ hybridization (FISH), in which fluorescently labeled probes target DNA and RNA in fixed cells, has limited resolution and can detect a small number of probes, super-resolution microscopy in combination with multiplexed probes allows for visualization of interactions involving >1000 genomic loci, including those located within a proximity of 10–100 kb^16,17^. By separating fluorescent signals in time that are too close to resolve in space, advanced microscopic techniques such as STORM/PALM overcome the diffraction limit to increase resolution^9^. Limitations on the number of genomic loci that can be visualized simultaneously have also been overcome by barcoding and sequential, combinatorial labeling approaches, including OligoFISSEQ^18^ and MERFISH (Fig. 1b)^17^. An analysis of chromosome 21 by MERFISH revealed a high correlation with previously published Hi-C data for various features of 3D genomic structure, including the distribution of A/B compartments as well as the location of TADs and TAD boundaries, despite substantial heterogeneity at the single-cell level^17^. MERFISH has been further adapted to image particular epigenomic features in a high-throughput manner by inclusion of TN5-mediated tagmentation targeting H3K4me3, H3K27ac, or H3K27me3 histone modifications that mark active promoters, active genomic loci, and silenced genomic loci, respectively. Epigenetic regions of interest were tagged with the T7 promoter for amplification of the targeted DNA fragments by in situ transcription, and the resulting RNAs were detected via MERFISH, revealing hundreds of genomic loci decorated with the specific histone marks^19^. This epigenomic MERFISH study showed the spatial distribution of putative enhancers, enhancer–promoter pairs, and enhancer hubs during mouse brain development. To benchmark the modified technique, images for a subset of labeled promoters were compared to the expression patterns of the corresponding genes reported in the Allen brain in situ hybridization (ISH) atlas, which demonstrated high congruence.

Live-cell imaging has extended an understanding of 3D genome structure to a fourth dimension by recording interaction dynamics over time. Visualization of chromatin loci in live cells can be achieved by imaging repeated sequences of DNA or RNA bound by fluorescently- labeled, sequence-specific nucleic acid binding proteins. Bacterial operon systems, such as the lactose operon (LacO/LacR), tetracycline operon (TetO/TetR) and cumate gene-switch (cuO/CymR), have been employed to visualize targeted genomic loci via insertion of multiple copies of an operator sequence and expression of the cognate repressor protein with a fluorescent tag. These systems can be combined for visualization of two different genomic loci with distinct fluorophores^20^. MS2 and its analog PP7, which are derived from RNA bacteriophages, are currently the most widely used RNA-imaging system. MS2 and PP7 sequences recruit their respective binding proteins, MCP and PCP, which can be differentially labeled by fluorescent protein fusion to enable dual-color, live tracking of RNA dynamics (Fig. 1c). The MS2/PP7 system also allows for detection of genomic loci that are actively transcribed. Accordingly, MS2/PP7 live-imaging studies have visualized EPIs and demonstrated the critical role of enhancers in controlling the transcriptional bursting dynamics of cognate gene promoters^21–23^. While targeting exogenous DNA or RNA sequences can confer robust signal strength, it is technically challenging due to the laborious nature of the requisite genetic manipulation(s).

CRISPR-based technologies, which allow for sequence-specific recognition without genetic editing, offer an alternative strategy that can be employed independently or in combination with MS2/PP7 labeling. An enzymatically-dead version of CRISPR-associated protein 9 (dCas9) has been used in imaging studies of native genomic regions to evaluate EPI dynamics. In this approach, signal amplification and/or multiplexed gRNA delivery is typically needed to improve the signal-to-background ratio and overcome the inefficient labeling of nonrepetitive genomic loci^24^. The discovery of the Cas13 family, comprising Cas13a-d, X, and Y, which specifically target RNA, has enabled the introduction of additional experimental tools that can be applied to EPI studies^25^. Recently, several reports have demonstrated the utility of enzymatically-dead CRISPR-Cas13 (dCas13) proteins as a new platform for RNA imaging in live cells^26^. Dual-color imaging has been achieved by employing orthologous dCas13 proteins and by coupling the dCas13 system with either MS2 RNA labeling or dCas9 genomic locus detection^27,28^. Although dCas13-mediated visualization of eRNAs has not been reported, this potential application would be valuable for further elucidation of the molecular underpinnings of EPI dynamics.

Methods for imaging 3D genome structure have been empowered by the development of super-resolution fluorescence microscopy technologies, especially single-molecule localization microscopy, such as SIM, SMLM, and STED, which have improved resolution to the low-nm range^29^. A technique called MINFLUX, which offers a superior resolution of 1–3 nm in fixed and live cell imaging, was recently combined with DNA-PAINT for multicolor labeling of mitochondria^30^. MINFLUX has not been applied to studies of genome architecture, but this nanoscopy approach may eventually yield additional insights into the molecular details of EPIs.

### Functional validation of enhancers

While strategies that provide 3D genome information and decipher the epigenetic landscape are useful for evaluating presumed CREs and their interactions, these approaches cannot validate enhancer activity in the native chromatin context. Genome editing techniques have been used to delete or alter certain enhancers in cell lines and animal models, but traditional approaches are laborious and low-throughput. Development of CRISPR-based, epigenome-editing technologies has allowed for relatively simple targeting of transcriptional repressive machinery to specific regions of interest, including enhancers and promoters, by coupling dCas9 to different repressors/repressor domains, such as the KRAB domain, methyl-CpG binding protein 2 (MeCP2), and DNA methyltransferases (DNMTs) (CRISPRi). Alternatively, dCas9 can be outfitted with activator domains to achieve the opposite effect (CRISPRa). KRAB repressor domain-fused dCas9 efficiently silences 200–500 base pair (bp) genomic regions in a sequence-specific manner by promoting deposition of suppressive histone modifications that induce heterochromatin formation via the recruitment of additional factors, such as KAP1 and HP1. The repressive capacity of dCas9-KRAB is enhanced by attachment of MeCP2 (dCas9-KRAB-MeCP2). In the case of DNMT fusions, DNA hypermethylation of target regulatory regions causes transcriptional down-regulation. Furthermore, when dCas9 is connected to KRAB, Dnmt3A, and Dnmt3L simultaneously, the system can confer a heritable repressive effect that persists even after cell division and differentiation (CRISPRoff)^31^. These CRISPR tools allow for validation of functional enhancers of particular target genes in a case-by-case fashion, but they also have been employed for multiplexed and genome-wide interrogation of enhancer function^32,33^. For example, a CRISPRi approach integrated with FISH and flow cytometry, called CRISPRi-FlowFISH, was used to identify functional noncoding variants from genome-wide association studies (GWASs)^34^. In addition, an integrative method termed STING-seq, which combines CRISPRi-based perturbation of GWAS-linked CREs and single-cell RNA sequencing, allowed for identification of causal variants that affect disease-related gene expression in a high-throughput manner^35^.

## Molecular mechanisms underlying EPI specificity

Although the role of enhancers in the regulation of gene transcription is well established, the fundamental question of how enhancers communicate with their distal gene promoter target(s) remains largely unresolved. In this section, we describe several molecular mechanisms that contribute to the enhancer–promoter interactome.

### Looping-dependent models of EPI

CTCF binding and loop extrusion by the molecular motor cohesin create a 3D framework for functional networks of dynamic long-range interactions between promoters and enhancers in the nucleus^7,36^. In this model, the DNA loop-extruding movement of cohesin along chromatin is halted at CTCF binding sites (CBSs) positioned in a convergent orientation. A higher frequency of physical interaction within the region demarcated by convergent CTCF sites, as measured by Hi-C, constitutes the basis of TADs or loop domains. Flanking CTCF sites serve as loop anchors/TAD boundaries that often have an insulator function (for a detailed review, see refs. ^37,38^). Targeted degradation of the cohesin machinery or CTCF compromises local TAD structure and TAD insulation, while higher-order chromatin structures (i.e., chromosome compartments) are not necessarily affected^39–41^. Conversely, transcription enforces TAD strength, long-range interactions and boundary site insulation^42^, possibly through weak CTCF-RNA interactions, biomolecular condensate formation, and/or the process of transcription itself^43,44^.

**Fig. 2:**
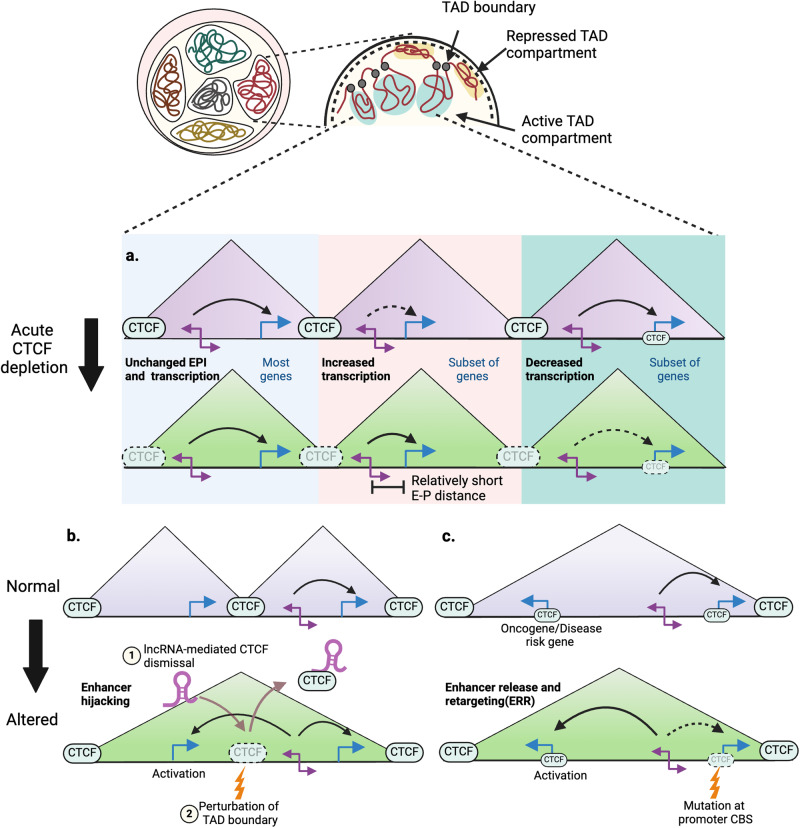
Effects of CTCF loss on enhancer–promoter interactions. **a** The effect of acute CTCF/cohesin depletion on transcription and EPI is remarkably limited. Despite widespread loss of chromatin loops anchored at CBSs and weakening of chromatin domain borders, only subtle changes in gene expression are detected. A subset of genes with increased transcription after CTCF degradation is characterized by relatively close positioning of its promoters to enhancer elements, while the other subset, comprising downregulated genes, shows loss of promoter-bound CTCF. **b** Two nearby TADs can combine in the absence of CTCF binding at a TAD boundary due to mutation, epigenetic perturbation of the CBS, or release of CTCF by RNA interaction. TAD fusion can lead to ectopic contact between promoters and enhancers, which is known as enhancer hijacking. **c** Genetic variants located in promoter CTCF binding sites prevent interaction of an enhancer with its preferred promoter, allowing the enhancer to interact with and activate other gene promoters in the same TAD, a phenomenon referred to as enhancer release and retargeting (ERR).

While discrepant results have emerged from degron-targeted CTCF degradation in cultured cells, such depletion experiments have generally revealed a surprisingly subtle immediate impact on transcription, affecting only a limited subset of genes initially despite the near complete loss of TADs (Fig. 2a)^41,45,46^. This may be attributed, at least in part, to the extent of CTCF protein degradation that is achieved by a degron system within a particular cell type. Although the degron approach routinely renders CTCF undetectable in nuclear lysates and even in chromatin fractions by immunoblotting, the amount of residual CTCF at individual binding sites can be highly variable, with some retaining 90% of CTCF binding upon acute depletion^47,48^. These differences may result in temporally nuanced outcomes for transcriptional regulation^41,49^, as not all CTCF binding sites are targeted with the same kinetics^48^. Stronger CTCF binding sites, which are functionally better insulators, seem to retain CTCF longer than weaker sites^41,48,50,51^, perhaps reflecting slower turnover of CTCF at the former or the effect of chromatin-based differential accessibility on the degron system. These findings also raise the possibility of context- and cell type-specific roles for CTCF in gene expression. However, limited transcriptional changes were observed in mouse embryonic stem cells (ESCs), even in the context of almost 100% degron-mediated depletion of CTCF or cohesin genome-wide at an early (3 h) time point^46^. Thus, a “molecular memory,” mediated by various chromatin features, might render CTCF, cohesin, and other looping factors dispensable for established EPI at least initially and buffer against any immediate transcriptional changes. This possibility is consistent with micro-C data demonstrating that global EPIs are largely maintained upon acute depletion of CTCF, cohesin or the looping-associated chromatin protein Ying Yang 1 (YY1), although they may weaken over time^46^. While nonessential for enhancer–promoter pairs that are closely positioned, more distant interactions rely increasingly on loop extrusion by cohesin or nearby boundary CTCF sites^51,52^. In addition, application of a tiled micro-C approach, allowing for even finer resolution of sub-TAD scale alterations, has revealed a decreased efficiency in EPIs and associated subtle transcriptional effects upon degron-mediated removal of cohesin. In this setting, weakened EPIs presumably persevere due to DNA contacts that involve transcription factors or CTCF via cohesin-independent looping mechanisms^39,53^.

Deletion of CTCF binding sites in TAD boundaries results in local transcriptional dysregulation due to altered EPI^54^, whereas complete genetic knockout (KO) of CTCF at the organismal level causes widespread changes in gene expression in addition to reduced TAD insulation. The latter manipulation was accomplished in zebrafish, which, unlike mice, can seemingly tolerate loss of endogenous CTCF until a relatively late stage of development due to the provision of maternal CTCF protein^55^. Among the limited set of genes that is differentially expressed after degron-mediated depletion of CTCF, upregulated genes tend to be located closer to enhancers^41,56^, likely indicating their activation by ectopic enhancer–promoter contacts that are also enabled by disruption of TAD insulation^45,53^. Genes that are down-regulated after CTCF degradation tend to display loss of CTCF binding in their promoters^41,45,47,49,56^, potentially reflecting the role of promoter-bound CTCF in chromatin accessibility and/or in enabling EPI (Fig. 2a)^47,56,57^.

**Fig. 3:**
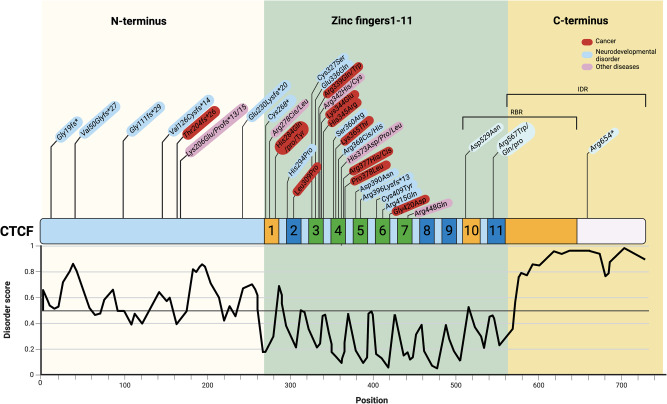
Schematic diagram of CTCF domain structure and the distribution of various disease-associated mutations. CTCF is characterized by a central 11 zinc-finger (ZnF) DNA-binding domain. ZnF3-7 (green) mediate binding to the core CTCF DNA motif. The IUPred3 disorder plot^168^ indicates the presence of an intrinsically disordered region (IDR) in the C-terminus. ZnF1, ZnF10, and part of the IDR domain are involved in RNA binding (orange). The relative position of a subset of documented disease-linked CTCF mutations is also indicated, with those implicated in neurodevelopmental disorders and diverse cancers colored in blue and red, respectively^132,134,135,169–172^.

Like many other chromatin-associated proteins, CTCF is thought to be functionally regulated by RNA interactions. CTCF zinc finger (ZnF) 1, ZnF10, and a C-terminal RNA-binding region have been shown to bind a number of lncRNAs with low affinity (Fig. 3)^58–62^, and modeling suggests the presence of shared structural motifs in the RNA interaction partners^63^. RNA binding contributes to CTCF self-association and its sub-nuclear dynamics^58–60,64^. In addition, genome-wide CTCF occupancy is altered by mutations that disrupt CTCF-RNA binding^59,60^, raising the intriguing possibility that CTCF-RNA interactions also shape the 3D genome. Notably, the X chromosome-encoded lncRNA Jpx has been reported to function as a CTCF release factor. Jpx selectively dismisses CTCF from loop anchor regions, which alters TAD structures and concomitantly impacts transcription due to newly formed EPIs (Fig. 2b)^61^. Thus, the emerging CTCF-RNA interactome might influence local interactions as well as global nuclear organization by fine-tuning TAD architecture. Disruption of these looping events by non-coding SNPs/mutations that affect expression or secondary structure of CTCF-interacting RNAs may have phenotypic consequences as a result of altered EPIs and consequent gene expression changes. However, it should be noted that the in vivo specificity of RNA interactions involving numerous chromatin-associated proteins, including CTCF and YY1, has recently been challenged, possibly necessitating further validation to confirm functional significance in many cases^65^.

Signal-induced nuclear receptor activation showcases the specificity of EPIs^66–68^. In the well-studied glucocorticoid receptor (GR) system, NIPBL is employed by GR to facilitate enhancer–promoter looping^69^. EPI specificity in estrogen receptor (ER)-dependent transcription is mediated by TF binding to enhancers that encourages condensate formation, the presence of cohesin, and the activity of topoisomerase I^7,70,71^. Nuclear hormone receptors themselves have been suggested to serve as anchors for intra-TAD looping^72^. The Mediator complex, which physically links nuclear hormone receptors and other transcriptional activators to the basal transcription machinery, also functionally connects enhancers to promoters^73^, although its architectural role in EPIs might not be direct and may involve the formation of condensates^74,75^.

### Looping-independent models of EPI

While genome-wide analyses have demonstrated that eukaryotic transcription arising from promoters and enhancers is subject to common regulatory controls that frequently involve their physical interaction or spatial proximity, looping-independent mechanisms of enhancer–promoter interplay are not mutually exclusive and may even prevail in some instances. Alternative models of EPI that do not necessarily require looping events include tracking, whereby enhancer-bound RNA Pol II scans intervening DNA in a linear fashion to seek out its cognate promoter, and linking, which entails oligomerization of a regulatory protein that binds interacting CREs to establish a linkage^76^. In the scanning model, RNA Pol II, if not continuously transcribing, might be aided by a DNA motor protein, such as cohesin, possibly helping to explain the effects of CTCF-bound insulator elements that, when situated intermediately within TAD boundaries or otherwise, restrain the capacity of enhancers to activate neighboring promoters^76,77^. The linking model derives from the observation that oligomerized bacteriophage lambda repressor connects bound regulatory sites, or operators, with target promoters^78^, but there is limited supporting evidence for this phenomenon in eukaryotic systems^76^.

Non-looping models of EPI do not stipulate increased 3D colocalization of regulatory elements. In this regard, super-resolution imaging of sonic hedgehog gene (*Shh*) activation in the context of neural differentiation has revealed decreased enhancer–promoter proximity that can be disrupted by DNA-tethered protein impediments^79^. The increased enhancer-promoter spatial separation was ascribed to the enzymatic activity of the transcriptional cofactor poly(ADP-ribose) polymerase 1 (PARP1), which synthesizes long, branched chains of the nucleic acid PAR as a covalent modification of histones and other proteins to promote chromatin decompaction and non-covalent protein recruitment. These observations are consistent with a tracking or linking model^79^. However, a different developmental enhancer seems to rely on looping-dependent spatial proximity, albeit largely invariant, to achieve appropriate spatiotemporal *Shh* expression^80^, and other examples in which enhancer–promoter distance shows a positive correlation with gene activation have not been rigorously characterized. Nevertheless, it is plausible that for some instances in which looping mediates EPI, linking and/or tracking mechanisms contribute to the physical or functional interplay of regulatory elements brought into spatial proximity^76^.

## Transcriptional control involving EPI

### Bursting

**Fig. 4:**
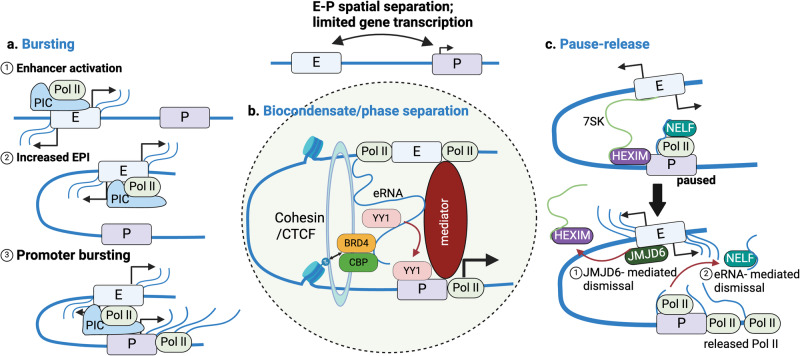
Transcriptional control involving enhancer–promoter interactions. **a** Gene expression occurs in a discontinuous fashion, referred to as bursting. Enhancers can increase bursting frequency to augment cognate gene expression via increased enhancer–promoter contact. Enhancer bursting, unlike promoter bursting, and synchronization of enhancer/promoter activation have yet to be visualized by imaging techniques. **b** Interaction of IDR-containing proteins (TFs, cofactors, and other chromatin-associated proteins) and their association with eRNAs results in formation of biocondensates. These phase-separated structures are thought to form at specific genomic loci to facilitate robust transcriptional activation. **c** Multiple mechanisms may allow enhancers to induce pause release at promoters by discharging pausing factors. Anti-pause enhancers (A-PEs) increase gene expression from cognate promoters via JMJD6-dependent dismissal of 7SK snRNA and HEXIM1/2. Alternatively, eRNAs can cause disassociation of promoter-bound NELF.

Expression of most, if not all, genes in prokaryotes and eukaryotes occurs in a discontinuous fashion, involving episodes of activity separated by refractory intervals of complete or near-complete inactivity. In metazoans, this pulsatile nature of gene expression, dubbed transcriptional bursting, may be crucial for regulation of developmental programs as well as for signal-dependent responses in differentiated cells or tissues. Mechanistically, transcriptional bursting is regulated by EPI as well as other DNA sequence and chromatin features known to impact gene expression, such as core promoter elements^81^, nucleosome positioning/density^82^, TF occupancy/dwell time^82^, and epigenetic modifications^21,83–85^. The frequency of bursting, as opposed to the amplitude or duration, is the parameter most acutely linked to enhancer activity (Fig. 4a)^81,85^. Strong enhancers increase bursting frequency at their cognate promoters, which corresponds to higher levels of gene expression^81,85^. Moreover, an artificially imposed linkage of the β-globin gene promoter with its distal enhancer results in transcriptional activation by selectively increasing bursting frequency^21^.

Bursting was first observed more than four decades ago in electron micrographs as ribonucleoprotein (RNP) fibers emanating in a discontinuous pattern from *Drosophila melanogaster* sister chromatids. In recent years, increasingly sophisticated techniques, including live cell imaging of nascent transcripts, single-molecule FISH (smFISH), and sc- or snRNA-seq, have been employed to study the phenomenon at static time points and in real time^86^. As the field continues to develop, there are still many unanswered questions, especially regarding EPIs. While Hi-C data indicate that EPI reliably predicts gene activation^87^, how activation of one or more enhancers, based on eRNA production, is coordinated with that of a target gene requires further inquiry. As non-coding transcription is also presumed to be discontinuous, it remains unclear to what extent bursting at promoters and enhancers is synchronized, although the latter is more challenging to investigate for biological as well as technical reasons. In addition, a biochemical dissection of the requisite components and molecular mechanism(s) governing bursting kinetics at promoters and enhancers in the context of EPIs, as might be feasible with cell-free assays^88,89^, would be particularly instructive.

### Pause release

Key points of regulatory control of promoter-driven transcription include recruitment of the pre-initiation complex (PIC), comprising general transcription factors as well as RNA Pol II, and subsequent release of proximally paused RNA Pol II^90^, both of which may be subject to modulation via EPI^88^. A study using yeast nuclear extracts and an upstream activating sequence (UAS)-promoter system showed the spatiotemporal dynamics of enhancer-promoter interplay in PIC assembly by single-molecule visualization^91^, suggesting sequential enhancer-promoter activation. In this context, RNA Pol II pre-assembles at the UAS/enhancer with other PIC components before the complexes are transferred to the core promoter. Similarly, at the α-globin gene locus in differentiating erythroid cells, the PIC is first recruited to distal enhancers prior to its promoter deposition in concert with increased enhancer–promoter association^92^. ChIP-seq assays have substantiated the widespread enhancer presence of the PIC^93^ along with that of the large Mediator coactivator complex^94^ in metazoans^74^. Although 3C assays indicate that Mediator can collaborate with cohesin to facilitate EPI^94^, promoter-based capture Hi-C data suggest that this may not be a global phenomenon^95^. Similarly, the role of the PIC in EPI might be limited globally^95^. Further genome-wide interrogation of various cell types and/or conditions is needed to elucidate any potential reciprocal relationship between RNA Pol II transcriptional machinery loading and EPI.

Following initiation, RNA Pol II typically transcribes less than 100 nucleotides before pausing, which is mediated by the pausing components DRB sensitivity-inducing factor (DSIF) and negative elongation factor (NELF). Paused RNA Pol II is released by the kinase activity of positive transcription elongation factor b (P-TEFb), which phosphorylates DSIF and NELF, altering the suppressive function of the former and causing dissociation of the latter to allow for resumption of transcription^96^. In multiple cell types, the arginine demethylase jumonji C-domain-containing protein 6 (JMJD6) and bromodomain-containing protein 4 (BRD4), an epigenetic reader of acetylated histones, collaborate at a collection of distal CREs, termed anti-pause enhancers (A-PEs), to facilitate pause release and thus gene activation at a large group of regulated promoters. Detailed mechanistic analysis supports a model in which JMJD6 is recruited to A-PEs by BRD4 to demethylate both 7SK snRNA and the modified histone with which it associates, H4R3me^2(s0)^. Accordingly, JMJD6 secures the dismissal of 7SK snRNA and HEXIM1/2, which collaborate to inhibit P-TEFb-mediated pause release (Fig. 4c). A-PEs associate with target gene promoters, as demonstrated by 3 C analysis, suggesting that EPI confers specificity in this regulatory strategy. Alternatively, EPI may also ensure local accumulation of *trans*-acting eRNAs, which can bind and dissociate NELF from regulated promoters to elicit pause release^97^. Notably, pausing factors, including NELF^94^ and PAF1^98^, bind to enhancers, and transcriptional pausing has been detected at enhancers genome-wide (Fig. 4c)^99^, but it is unknown whether pause release at functionally linked enhancers and promoters occurs in synchrony via their interaction.

### Phase separation

Recent appreciation of the potential biological role(s) of condensates formed by liquid‒liquid phase separation (LLPS) has altered views on the formation and function of membraneless structures within cells, such as nucleoli, nuclear speckles, and stress granules, while affording new insights into many cellular activities, including transcription^71,100–105^ as well as dynamic changes in 3D chromatin architecture^43,103^. The activation domain of TFs often features one or more intrinsically disordered regions (IDRs), stretches of amino acids, sometimes with low sequence complexity, that do not fold into stable structures but rather can assume various conformations and bind diverse partners. CTCF and other architectural proteins are also predicted to contain IDRs (Fig. 3), which may have functional implications for the (re)organization of genome topology. IDR-containing proteins tend to self-associate and interact with each other via numerous IDR-mediated, low-affinity, multivalent interactions^106^. Depending on their sequence composition, many IDRs can also bind RNA, either alone or in collaboration with RNA-binding motifs that are frequently found in the same protein^107^. Accordingly, formation of condensates, which are nucleated by enhancer sequences that attract various IDR-containing TFs and induce short promoter-derived ncRNAs that act in concert with eRNAs, has been proposed to facilitate transcriptional initiation (Fig. 4b)^101,102,108^. However, high levels of RNA produced by elongating RNA Pol II may promote condensate dissolution via a negative feedback effect, possibly resulting in the refractory period that follows a transcriptional burst. Thus, LLPS offers a potential biophysical explanation for the pulsatile nature of transcription promoted by EPI^101,102^. Condensate formation may also help to explain the coordinated bursting of two genes by a single enhancer^85,109^ as well as cooperative chromosomal enhancer assembly^71^. However, while in vitro assays using purified proteins have allowed for exquisitely detailed analysis of the biochemical determinants of condensate formation as well as their functional potential, the tools available for studying phase separation within intact cells remain limited, and, consequently, definitive proof of its role(s) in transcriptional regulation and genome structural organization has not been achieved^110^.

## EPIs in disease

Changes in enhancer function or activity with pathological consequences have been ascribed to multiple molecular mechanisms, including SNPs, epigenetic modifications, genomic structural variants (Table 1), and mutations in architectural proteins (Table 2). These ‘enhanceropathies’ clearly involve altered EPI in some instances, but this aspect has not been rigorously investigated in many cases.

**Table 1:** Alterations in enhancer function impacting human pathology.

Gene	Genomic location	Variants	Molecular mechanism	Disease association	Refs.
RASGRP1	15q14	GWAS SNP	Loss of enhancer activity	autoimmunity	^34^
RET	10q11.2	GWAS SNP	Loss of enhancer activity	Hirschsprung’s disease	^173^
PITX2	4q25	GWAS SNP	Loss of enhancer activity	atrial fibrillation	^174^
TRIM46	1q22	GWAS SNP	Gain of enhancer activity	breast cancer	^175^
CEACAM21	19q13	GWAS SNP	Gain of enhancer activity	prostate cancer	^176^
ZFP90	16q22.1	GWAS SNP	Gain of enhancer activity	colorectal cancer	^177^
LMNB1 (lamin B1)	5q23.2	0.6 Mb deletion	TAD fusion; LMNB1 activation by 3 enhancers from neighboring TAD	autosomal dominant adult-onset demyelinating leukodystrophy (ADLD)	^178^
WNT6, IHH, EPHA4, PAX3	2q35–36	deletions (1.75–1.9 Mb), heterozygous inversions (1.1–1.4 Mb), duplications (0.9–1.2 Mb)	Ectopic expression due to *Epha4* enhancer hijacking resulting from deletions (PAX3) or inversions/duplications (WNT6/IHH) affecting TAD boundaries	limb malformation (brachydactyly, syndactyly/ F-syndrome, polydactyly)	^179,180^
HBB (β-globin)	11p15.4	CTCF site inversion	neo-TADs formed by convergent CTCF sites; enhancer hijacking	β-thalassemia	^181^
PDGFRA	4q12	CTCF site hypermethylation (IDH gain of function); CTCF site deletion	loss of insulation between topological domains; TAD fusion	glioma	^182^
TAL1, LMO2	1p33, 11p13	CTCF site deletion	loss of insulation; proto-oncogene activation from new enhancers	T-cell acute lymphoblastic leukemia (T-ALL)	^183^
EVI1/GATA2	t(3;3)(q21;q26.2)	GATA2 enhancer repositioning ectopically activates EVI1	enhancer hijacking	acute myeloid leukemia (AML)	^184^
GFIB	9q34.13	deletion, inversion, complex rearrangements	local enhancer hijacking	medulloblastoma	^185^
GFI	1p22.1 → t(1:6) and t(1:9)	translocations	‘distal’ enhancer hijacking following translocation	medulloblastoma	^185^
NOTCH1	9q34.3	CTCF site mutation	possible TAD disruption	ovarian cancer	^186^
CCNE1 (cyclin E1)	19q12	CTCF site mutations	possible TAD disruption	liver, ovarian, pancreatic cancer	^186^
GDPD1	17q22	deletions, complex rearrangements (multiplications, inversions) at a TAD boundary	local enhancer hijacking after neo-TAD formation	autosomal-dominant retinitis pigmentosa (adRP)	^187^
TFAP2A (AP-2α)	inv(6)(p24.3;q16.2)	89 Mb inversion	enhancer disconnection	branchiooculofacial syndrome (BOFS)	^188^
PAX3:FOXO1	t(2;13)(q35;q14)	translocation	‘distal’ enhancer hijacking by the fusion gene after translocation	alveolar rhabdomyosarcoma	^189^
NTS (neurotensin)	12q21.31	gained CTCF promoter binding that enables enhancer-promoter looping	enhancer hijacking	uveal melanoma	^190^
NR0B1 (DAX1)	Xp21.2	tandem duplication (1.2 Mb), inversions	neo-TADs; TAD shuffling leading to enhancer hijacking	46,XY gonadal dysgenesis	^191^
FMR1 (FMRP)	Xq27.3	expansion of short tandem repeats	boundary disruption resulting in enhancer disconnection	fragile X syndrome	^192^

A non-exhaustive list of studies implicating altered enhancer function in human disease-relevant gene expression changes. (For a detailed discussion, see refs. ^112,193–196^).

**Table 2:** Architectural proteins and their disease associations in humans.

Protein/Protein complex	Disease association	Mutation	Molecular mechanism	Refs.
CTCF	breast, kidney, prostate cancer; leukemias; endometriosis; endometriosis-associated neoplasia	CTCF copy number aberrations, N-terminal mutations p.Gly19*, pThr204fs*26, K206E, and DBD (ZnF1-5) mutations R278C, H284N/P/Y, L309P, R339Q/W, R342H, K344E, H345R K365T, H373L, R377C/H, P378L, R448Q; also see Fig. 3	changes in CTCF protein levels; altered CpG methylation patterns; modified CTCF DNA-binding profile	^131,134,135,171,172,197,198^
	neurodevelopmental disorders; cardiac defects	numerous DBD and non-DBD frameshifts, including p.Gly111fs*29, pVal126Cysfs*14, p.Lys206Profs*13/15, pArg396Lysfs*13; missense mutations in DBD ZnF (e.g., D390N, R567W); also see Fig. 3	altered CTCF binding or not determined	^130,132,169,170,197,199^
	multiple cancer types; neurodegenerative disease; severe influenza; hearing loss; osteoporosis	CTCF binding site mutations	loss of CTCF binding	^118,130,136,137^
Cohesin	Cornelia de Lange syndrome	NIPBL, SMC1A, SMC3, RAD21 Indel, frameshift, missense mutations	multiple, including defective DNA repair, chromosome instability, and disruption of TADs/E-P interactions	^140,141^
	peripheral sclerocornea	RAD21 A622T	dysregulated cohesin binding	^200^
	chronic intestinal pseudo-obstruction/Mungan syndrome	RAD21 R450C	separase cleavage site in RAD21	^201^
Condensin	neurodevelopmental disorders	NCAPH, NCAPD2 missense and splice site mutations; NCAPD3 frameshift, intronic mutation creating a de novo splice site	impaired chromosome segregation and chromosome structural integrity	^148^
	neurological disorders, nervous system tumors	NCAPH2, NCAPG2, NCAPD2, NCAPH deletion and haploinsufficiency; SMC2, SMC4, NCAPG2 overexpression	multiple, including defective DNA repair and TGFβ pathway activation	^147^
MeCP2	Rett syndrome	deletions and missense mutations, predominantly in methyl-binding and corepressor interacting domains	transcriptional derepression; loss of repressive chromatin looping	^157,159,202^
YY1	neurological diseases, intellectual disability syndrome	deletions and missense mutations resulting in haploinsufficiency	loss of YY1 binding at low occupancy sites; reduced H3K27ac on cognate YY1 enhancers	^156,203^

A survey of disease-related mutations in selected proteins that are important for 3D genomic architecture.

### Enhancer variants

Small insertions (1–31 bp), which are readily detected in cancer cell genomes, can alter enhancer activity or potentially generate de novo enhancers, leading to transcriptional changes that can affect oncogene expression^111^. Disease-linked SNPs detected by GWASs are predominantly located in non-coding sequences, including intronic and intergenic regions that often constitute enhancers; however, enhancer–promoter pairs require experimental identification/validation, and disease-relevant enhancers typically show cell type-specific activity, complicating straightforward pathological assignments for SNPs (as well as de novo mutations) found in putative enhancers.

In addition to genetic perturbations, epigenetic changes can impact enhancer activity/function with direct or indirect effects on EPIs. In this regard, enhancer-specific alterations in DNA methylation or in the histone modification landscape, including H3K27ac profiles, have been linked to specific disease phenotypes (Table 1)^112^. Comprehensive analysis of enhancer activity based on DNA hypersensitivity and H3K27ac for almost 9000 samples representing 33 cancer types revealed genome-wide activation of enhancers accompanied by tumor aneuploidy^113^, suggesting that enhancer activity not only promotes oncogene expression but also facilitates chromosomal structural rearrangements. Human endogenous retroviral loci constitute a potential alternative source of enhancer activity impacting proximal gene promoters in cancer^114^. Reactivation of human endogenous retrovirus (HERV) subfamily K was recently shown to contribute to senescence-associated inflammation and aging^115^, indicating even broader (patho)physiological relevance, but the role of an HERV-derived enhancer program as well as EPI in adjacent and distal gene expression requires further investigation in diverse cell types. Notably, CRISPRi strategies, which feature high specificity and scalability, have greatly enabled identification of functional EPIs and evaluation of enhancer epigenomic landscapes in looping events^32–35^.

### Enhancer mistargeting by alteration of 3D genomic organization

Changes in 3D genome structure can cause alterations in EPIs that induce expression of genes other than the original target(s). Genomic rearrangements, which are commonly observed in most cancer types and sometimes may arise from a single catastrophic event^116^, can reposition CREs to allow for enhancer hijacking, a scenario in which an enhancer regulates one or more new target gene promoters^112^. Copy number variation that alters TAD structure due to disruption of TAD boundaries, allowing for enhancer retargeting, has been implicated in a few congenital diseases and several cancers^112^. Analysis of structural variants (SVs) in more than 1200 cancer samples by whole-genome sequencing revealed hundreds of genes located ≤100 kb of an SV breakpoint with altered expression, most of which were up-regulated, including the cancer-associated genes TERT, MDM2, CDK4, ERBB2, CD274, PDCD1LG2, and IGF2^117^. Remarkably, while TAD disruption, which can also be caused by point mutations in boundary-localized CTCF-binding motifs^118^, may be a frequent feature of cancer genomes, its effects on proximal gene expression are usually limited^112,119^. Overall, 3D genome studies have convincingly implicated enhancer mistargeting and hijacking as disease mechanisms, but there are currently few validated examples due to the traditional challenges of identifying functional enhancers and determining their cancer-driving potential.

### Promoter variants

Alterations in promoter sequences can impact nearby gene expression beyond the associated gene body. Recent reports suggest that functional loss of a particular promoter due to mutation results in release of its cognate enhancer, which can then engage and activate alternative promoters in the same TAD by a mechanism called enhancer release and retargeting (ERR) (Fig. 2c)^57^. At the *TFF1* locus in MCF7 cells, deletion of the *TFF1* promoter, which is the preferred target of the proximal *TFF1* enhancer (TFF1e), allows for an ectopic looping event between TFF1e and a different promoter, namely the *TFF3* promoter that is located 50 kb away. This retargeting causes a > 20-fold increase in *TFF3* transcription. In addition to cancer-associated SNPs in the *TFF1* promoter that correlate with *TFF3* expression, activation of other proto-oncogenes, such as MYC, can be induced by alteration of neighboring gene promoters^57,120^.

Additional examples of ERR in cancer and development have been reported. In B-ALL, deletion of the *FLT3* promoter correlates with ectopic upregulation of the *CDX2* gene, which is positioned 30 kb away, and coincides with retargeting of an upstream enhancer to the promoter of the latter^121^. In gastric cancer, analysis of ENCODE and GTEx eQTL data revealed that a gene encoding the class A orphan GPCR protein GPR35 is activated by the ERR mechanism^122^. *GPR35* expression is induced by a retargeted upstream enhancer upon mutation of the CTCF-binding region in the promoter of the neighboring gene, *CAPN10*. During mesoderm formation, enhancer-dependent upregulation of *Mesp2* was observed in KO embryos for the nearby *Mesp1* gene^123^. Furthermore, promoter competition resembling ERR has been documented for the β-globin locus control region (LCR)^124^. In one scenario, deletion of the α-globin genes augmented expression of the *NME4* gene seemingly due to increased interaction of the latter with the major upstream regulatory element MCS-R2, despite a linear distance of 300 kb. However, it is unclear why ectopic contact with MCS-R2 was insufficient to elicit expression of several other genes in the region. At the same locus, impairment of the *HBB* promoter redirected the LCR from the adult *HBB* gene to the fetal *HBG* genes, resulting in increased production of fetal HBG^125^. ERR-like phenotypes are also observed during transvection, a phenomenon involving EPI between paired homologous chromosomes in *Drosophila*. In this context, several cases have been described in which deletion of a promoter for an allele on the same homolog as its enhancer results in increased promoter activity of the allele on the other chromosome^126^. Finally, a potential contribution of ERR in autism spectrum disorder (ASD) has recently been suggested. Rare promoter de novo variants (DNVs) identified by whole-genome sequencing of ASD individuals are enriched in TADs that contain known ASD risk genes but are often located in the promoters of constituent non-ASD risk genes. Transcriptomic analysis of modified iPSC lines harboring selected DNVs revealed gene dysregulation within the TAD as well as global expression changes, including that of multiple ASD risk genes. While these observations are suggestive of altered 3D genome architecture and CRE interactions in this neurodevelopmental disease setting, definitive proof of the ERR mechanism remains to be established^127^.

### The role of architectural proteins in disease-associated 3D genome structure

As a major regulator of 3D genome structure in most metazoans, CTCF influences gene expression under physiological and pathological conditions (Table 2)^128–130^. Haploinsufficiency in a *Ctcf*^+/-^ mouse model leads to increased tumorigenesis due to disrupted CpG methylation^131^. In humans, CTCF mutations have been implicated in various cancers and also contribute to neurological disorders^132–135^. CTCF mutations associated with human diseases are often located in the zinc finger (ZnF) DNA-binding domain (DBD), which can have pleiotropic effects as a result of altered transcriptional regulation and TAD organization (Fig. 3)^134,135^. In addition, CTCF binding site mutations are frequent in cancers^118,130,136–138^, leading to loss of insulator sites and aberrant transcription. When promoter CTCF sites are mutated, enhancer retargeting to neighboring gene promoters can occur^57^.

Somatic mutations in components of the cohesin complex have been reported in several cancer types whereas inherited mutations cause human developmental disorders that feature profound genome instability without any cancer predisposition (Table 2). The latter group, collectively referred to as ‘cohesinopathies’, includes Cornelia de Lange syndrome (CdLS)^139^. In a cell model of CdLS harboring a mutation in the cohesin-loading factor NIPBL, gene clusters with differential cohesin binding display disease-associated gene expression changes that may arise from disrupted promoter-promoter and enhancer-promoter contacts^140^. Induced proteolytic cleavage of the cohesin subunit RAD21 in postmitotic neurons recapitulates the transcriptional changes observed in CdLS patients^141^. In some solid and hematopoietic cancers, cohesin component expression levels correlate with prognosis and metastasis^142,143^. Although involvement of cohesin in cancer was initially ascribed to chromosome segregation defects caused by aberrant sister chromatid separation during the cell cycle and associated aneuploidy^143^, recent studies have demonstrated the relevance of its roles in TAD organization and transcriptional dysregulation. Cancer-associated mutations in cohesin subunits are overrepresented in STAG2^142^. Degron-mediated depletion of STAG2 in hematopoietic stem cells does not cause widespread disruption of chromosomal compartments or TADs, likely due to partial compensation by STAG1^144^. However, loss or downregulation of STAG2 specifically disrupts the local structure of a subset of TADs associated with stem cell self-renewal and differentiation, leading to the formation of new, long-range DNA loops to more distal sites and concomitant transcriptional dysregulation^144,145^, consistent with a tumor-suppressor role of cohesin in some cancers^146^.

Rare mutations in genes encoding components of condensin complexes have also been linked to human disease (Table 2). ‘Condensinopathies’ caused by inactivating mutations in condensin subunit genes typically manifest as neurodevelopmental disorders^147^. Mutations in the condensin II subunits NCAPD2, NCAPD3, and NCAPH result in loss of chromosome structural integrity and impaired chromosome segregation^148^. Cohesin and condensin have distinct spatially- and temporally-defined roles in loop extrusion during the cell cycle^37,38,149^, which is also the case for the two condensin complexes. Condensin II is located in the nucleus throughout the cell cycle, whereas condensin I is largely restricted to the cytoplasm during interphase^150^. In fission yeast, Hi-C interrogation has revealed chromosomal interactions that are quantitatively dependent on condensin, and degron-induced loss of condensin increases DNA mobility^151^. Similar to cohesin disruption, some condensin mutations may impact transcriptional activity as a result of alterations in local chromatin compaction and TAD structure.

The zinc-finger protein YY1 is a context-dependent activator/repressor that mediates EPI and selectively regulates pluripotency-related gene expression^152,153^. The ubiquitously expressed transcription factor may regulate as much as 10% of the human transcriptome^154^, and altered YY1 expression has been linked to various cancers and neurological diseases (Table 2)^155,156^. Since YY1 interacts with chromatin modifiers as well as chromatin-remodeling complexes and has roles in DNA repair, it remains largely unknown whether YY1 looping function is a salient feature of its diverse disease associations^155^.

MeCP2 is a ubiquitously expressed epigenetic regulator that is present at high levels in neurons and has been associated with multiple neurological disorders (Table 2). Notably, MeCP2 is mutated in 95% of individuals with Rett syndrome (RTT)^157^. Although MeCP2 can serve as a transcriptional activator in certain contexts, its role in inhibiting transcription via recruitment of the NCoR1/2 corepressor complex to specific sites of methylated DNA is crucially compromised in RTT^158^. As evidence of its relevance to disease-related genomic architecture, the RTT-associated Dlx5-Dlx6 locus is derepressed in Mecp2^-/-^ mice, and a repressive chromatin loop normally mediated by Mecp2 is replaced with longer-distance activating chromatin-associated loops^159^.

## Conclusion and outlook

Our understanding of enhancer function in gene expression has dramatically evolved with the increased capacity to probe and precisely perturb the 3D structure of the genome. Over 25% of sub-TAD cohesin-dependent chromatin loops are cell type-specific and tend to correlate, albeit weakly, with variations in gene expression between cell types^160^. Cohesin-mediated loops are enriched for enhancers, and cohesin-bound enhancers have a propensity to interact with other enhancers as well as TSSs^160^. These findings, published in phase III of the ENCODE project, underscore the importance of EPIs but also illuminate ongoing challenges, which largely pertain to the issue of identifying the functional enhancer(s) of a gene. Indeed, a given gene may have multiple enhancers of varying functional significance. While the search space for EPIs has been proposed to be limited to the size of a TAD (i.e., ~1 Mb), this restriction has been questioned. Moreover, a given enhancer does not necessarily contact the nearest gene promoter and may have limited or no functional significance to its neighboring gene even if there is evidence of looping^161^. Finally, some or all EPIs for a particular gene may be cell type specific. Despite this complexity, there are now a number of tools, including 3C-based and imaging modalities, that make it feasible to determine functional EPI for one or many loci when combined with CRISPR genome or epigenome editing.

The role of altered EPI in human disease phenotypes is becoming increasingly apparent, and multiple molecular mechanisms have been described. Changes in EPI due to structural variation in enhancers and promoters as well as mutations in the TFs and architectural proteins that associate with them, or alterations in their binding sites, have all been implicated as drivers of oncogenic transformation^112,162^. Furthermore, the preponderance of GWAS SNPs are located in non-coding intronic and intergenic regions that are putative enhancers or other regulatory sites^112^, consistent with the possibility that alterations in EPI contribute to a multitude of pathological conditions emanating from various tissues^112,163^.

While dysregulation of EPIs can cause transcriptional changes in disease, they also present a promising target for therapeutic interventions^112,163^. A few epigenetic therapies that may impact EPI are already clinically available, including hypomethylating agents (HMAs) that inhibit DNMTs and histone deacetylase inhibitors (HDACis), for treatment of specific hematological malignancies^11^. These drugs broadly affect the epigenomic landscape, which can be drastically altered in the context of malignant transformation due to chromosomal rearrangements or other genetic alterations in addition to the emergence of cancer-specific super enhancers (SEs). Cancer cells rely crucially on SEs that engage in EPIs to drive oncogenic transcriptional dysregulation^164^. Accordingly, epigenetic agents targeting various SE components, including bromodomain and extraterminal domain (BET) proteins such as BRD4, have been extensively evaluated as potential anticancer therapies, yet none have attained clinical approval. Broader application of these different classes of epigenetic drugs in cancer treatment may require multimodal regimens instead of their use as monotherapies^165^.

The limited specificity of traditional epigenetic drugs may restrict their therapeutic utility beyond cancer treatments. Ultimately, enhancer-based therapies using CRISPR-derived epigenome-modulating or base-editing tools will likely offer alternative options with unparalleled specificity for many conditions. The first CRISPR-based drug, dubbed Casgevy, which was approved for use in the United Kingdom in November 2023 and shortly thereafter by the U.S. Food and Drug Administration (FDA), actually targets an erythroid-specific *BCL11A* enhancer initially implicated in the control of fetal hemoglobin (HbF) levels by the presence of GWAS SNPs^166,167^. Cas9-dependent disruption of this enhancer element in isolated CD34^+^ hematopoietic stem cells causes marked downregulation of the BCL11A repressor and concomitant upregulation of its target genes, which include those encoding the γ-globin subunits, allowing for stable resumption of HbF production that is protective against sickle cell disease and β-thalassemia. Furthermore, multiple clinical trials using base editors to introduce specific genetic changes, without a requirement for DNA double-strand breaks, in cells ex vivo or following in vivo delivery are currently underway. These tools may eventually be employed for precise modification of other GWAS enhancer or promoter SNPs that contribute to disease susceptibility through altered EPI. It is also possible to envision future therapeutic applications of Cas13-mediated eRNA depletion as well as dCas9/12-imposed enhancer/promoter repression or activation in EPI-linked pathologies. Development of effective CRISPR-based therapies will benefit from integration of single-cell and spatial multiomic strategies, providing genomic, epigenomic, and transcriptomic data^167^, in studies of functional EPIs and the consequences of their perturbation. These approaches will also allow for systematic evaluation of seemingly rare CRISPR-related off-target effects, a lingering concern that is a particularly important consideration for treatments involving in vivo modification(s). Nevertheless, based on recent progress, therapies targeting disease-associated EPI are now within the purview of precision medicine.

## References

[1] Heintzman ND (2009). Histone modifications at human enhancers reflect global cell-type-specific gene expression. Nature.

[2] Andersson R, Sandelin A (2020). Determinants of enhancer and promoter activities of regulatory elements. Nat. Rev. Genet..

[3] Moore JE (2020). Expanded encyclopaedias of DNA elements in the human and mouse genomes. Nature.

[4] Preissl S, Gaulton KJ, Ren B (2023). Characterizing cis-regulatory elements using single-cell epigenomics. Nat. Rev. Genetm.

[5] Calderon D (2019). Landscape of stimulation-responsive chromatin across diverse human immune cells. Nat. Genetm.

[6] Dao LTM, Spicuglia S (2018). Transcriptional regulation by promoters with enhancer function. Transcription.

[7] Nair SJ (2022). Transcriptional enhancers at 40: evolution of a viral DNA element to nuclear architectural structures. Trends Genet..

[8] Consortium G (2020). The GTEx consortium atlas of genetic regulatory effects across human tissues. Science.

[9] Boettiger A, Murphy S (2020). Advances in chromatin imaging at kilobase-scale resolution. Trends Genet..

[10] Friedman MJ, Lee H, Kwon YC, Oh S (2022). Dynamics of viral and Host 3D genome structure upon infection. J. Microbiol. Biotechnol..

[11] Friedman MJ, Lee H, Lee JY, Oh S (2023). Transcriptional and epigenetic regulation of context-dependent plasticity in T-Helper Lineages. Immune Netw..

[12] Lafontaine DL, Yang L, Dekker J, Gibcus JH (2021). Hi-C 3.0: improved protocol for genome-wide chromosome conformation capture. Curr. Protoc..

[13] Brant L (2016). Exploiting native forces to capture chromosome conformation in mammalian cell nuclei. Mol. Syst. Biol..

[14] Rao SS (2014). A 3D map of the human genome at kilobase resolution reveals principles of chromatin looping. Cell.

[15] Quinodoz SA (2022). SPRITE: a genome-wide method for mapping higher-order 3D interactions in the nucleus using combinatorial split-and-pool barcoding. Nat. Protoc..

[16] Liu S, Zhao K (2021). The toolbox for untangling chromosome architecture in immune cells. Front. Immunol..

[17] Su JH, Zheng P, Kinrot SS, Bintu B, Zhuang X (2020). Genome-scale imaging of the 3D organization and transcriptional activity of chromatin. Cell.

[18] Nguyen HQ (2020). 3D mapping and accelerated super-resolution imaging of the human genome using in situ sequencing. Nat. Methods.

[19] Lu T, Ang CE, Zhuang X (2022). Spatially resolved epigenomic profiling of single cells in complex tissues. Cell.

[20] Alexander, J. M. et al. Live-cell imaging reveals enhancer-dependent. *Elife* 8 (2019). 10.7554/eLife.41769 10.7554/eLife.41769 PMC6534382 31124784

[21] Bartman CR, Hsu SC, Hsiung CC, Raj A, Blobel GA (2016). Enhancer regulation of transcriptional bursting parameters revealed by forced chromatin looping. Mol. Cell.

[22] Levo M (2022). Transcriptional coupling of distant regulatory genes in living embryos. Nature.

[23] Chen H (2018). Dynamic interplay between enhancer-promoter topology and gene activity. Nat. Genet..

[24] Van Tricht C, Voet T, Lammertyn J, Spasic D (2023). Imaging the unimaginable: leveraging signal generation of CRISPR-Cas for sensitive genome imaging. Trends Biotechnol..

[25] Bot JF, van der Oost J, Geijsen N (2022). The double life of CRISPR-Cas13. Curr. Opin. Biotechnol..

[26] Cao H (2022). Progress of CRISPR-Cas13 Mediated Live-Cell RNA imaging and detection of RNA-Protein interactions. Front. Cell Dev. Biol..

[27] Yang LZ (2019). Dynamic imaging of RNA in living cells by CRISPR-Cas13 systems. Mol. Cell.

[28] Wang H (2019). CRISPR-mediated live imaging of genome editing and transcription. Science.

[29] Jerkovic I, Cavalli G (2021). Understanding 3D genome organization by multidisciplinary methods. Nat. Rev. Mol. Cell Biol..

[30] Ostersehlt LM (2022). DNA-PAINT MINFLUX nanoscopy. Nat. Methods.

[31] Nuñez JK (2021). Genome-wide programmable transcriptional memory by CRISPR-based epigenome editing. Cell.

[32] Carleton JB, Berrett KC, Gertz J (2017). Multiplex enhancer interference reveals collaborative control of gene regulation by estrogen receptor α-bound enhancers. Cell Syst..

[33] Xie S, Duan J, Li B, Zhou P, Hon GC (2017). Multiplexed engineering and analysis of combinatorial enhancer activity in single cells. Mol. Cell.

[34] Nasser J (2021). Genome-wide enhancer maps link risk variants to disease genes. Nature.

[35] Morris JA (2023). Discovery of target genes and pathways at GWAS loci by pooled single-cell CRISPR screens. Science.

[36] Merkenschlager M, Odom DT (2013). CTCF and cohesin: linking gene regulatory elements with their targets. Cell.

[37] Higashi TL, Uhlmann F (2022). SMC complexes: lifting the lid on loop extrusion. Curr. Opin. Cell Biol..

[38] Davidson IF, Peters JM (2021). Genome folding through loop extrusion by SMC complexes. Nat. Rev. Mol. Cell Biol..

[39] Schwarzer W (2017). Two independent modes of chromatin organization revealed by cohesin removal. Nature.

[40] Rao SSP (2017). Cohesin loss eliminates all loop domains. Cell.

[41] Nora EP (2017). Targeted degradation of CTCF decouples local insulation of chromosome domains from genomic compartmentalization. Cell.

[42] Ulianov SV (2016). Active chromatin and transcription play a key role in chromosome partitioning into topologically associating domains. Genome Res..

[43] Gamliel A (2022). Long-distance association of topological boundaries through nuclear condensates. Proc. Natl. Acad. Sci. USA.

[44] Islam Z (2023). Active enhancers strengthen insulation by RNA-mediated CTCF binding at chromatin domain boundaries. Genome Res..

[45] Zhang H (2021). CTCF and transcription influence chromatin structure re-configuration after mitosis. Nat. Commun..

[46] Hsieh TS (2022). Enhancer-promoter interactions and transcription are largely maintained upon acute loss of CTCF, cohesin, WAPL or YY1. Nat. Genet..

[47] Hyle J (2019). Acute depletion of CTCF directly affects MYC regulation through loss of enhancer-promoter looping. Nucleic Acids Res..

[48] Luan J (2021). Distinct properties and functions of CTCF revealed by a rapidly inducible degron system. Cell Rep..

[49] Zuin J (2014). Cohesin and CTCF differentially affect chromatin architecture and gene expression in human cells. Proc. Natl. Acad. Sci. USA.

[50] Huang H (2021). CTCF mediates dosage- and sequence-context-dependent transcriptional insulation by forming local chromatin domains. Nat. Genet..

[51] Rinzema NJ (2022). Building regulatory landscapes reveals that an enhancer can recruit cohesin to create contact domains, engage CTCF sites and activate distant genes. Nat. Struct. Mol. Biol..

[52] Kane L (2022). Cohesin is required for long-range enhancer action at the Shh locus. Nat. Struct. Mol. Biol..

[53] Aljahani A (2022). Analysis of sub-kilobase chromatin topology reveals nano-scale regulatory interactions with variable dependence on cohesin and CTCF. Nat. Commun..

[54] Ushiki A (2021). Deletion of CTCF sites in the SHH locus alters enhancer-promoter interactions and leads to acheiropodia. Nat. Commun..

[55] Franke M (2021). CTCF knockout in zebrafish induces alterations in regulatory landscapes and developmental gene expression. Nat. Commun..

[56] Xu B (2021). Acute depletion of CTCF rewires genome-wide chromatin accessibility. Genome Biol..

[57] Oh S (2021). Enhancer release and retargeting activates disease-susceptibility genes. Nature.

[58] Hansen AS (2019). Distinct classes of chromatin loops revealed by deletion of an RNA-binding region in CTCF. Mol. Cell.

[59] Saldaña-Meyer R (2014). CTCF regulates the human p53 gene through direct interaction with its natural antisense transcript, Wrap53. Genes Dev..

[60] Saldaña-Meyer R (2019). RNA interactions are essential for CTCF-mediated genome organization. Mol. Cell.

[61] Oh HJ (2021). Jpx RNA regulates CTCF anchor site selection and formation of chromosome loops. Cell.

[62] He C (2016). High-resolution mapping of RNA-binding regions in the nuclear proteome of embryonic stem cells. Mol. Cell.

[63] Kuang S, Wang L (2020). Identification and analysis of consensus RNA motifs binding to the genome regulator CTCF. NAR Genom. Bioinform..

[64] Hansen AS, Amitai A, Cattoglio C, Tjian R, Darzacq X (2020). Guided nuclear exploration increases CTCF target search efficiency. Nat. Chem. Biol..

[65] Guo, J. K. et al. Denaturing purifications demonstrate that PRC2 and other widely reported chromatin proteins do not appear to bind directly to RNA in vivo. *Mol. Cell*. 10.1016/j.molcel.2024.01.026 (2024). 10.1016/j.molcel.2024.01.026 PMC10997485 38387462

[66] Yang F (2017). Glucocorticoid receptor:megatrans switching mediates the repression of an ERα-regulated transcriptional program. Mol. Cell.

[67] Johnson TA (2018). Conventional and pioneer modes of glucocorticoid receptor interaction with enhancer chromatin in vivo. Nucleic Acids Res..

[68] Warwick T, Schulz MH, Gilsbach R, Brandes RP, Seuter S (2022). Nuclear receptor activation shapes spatial genome organization essential for gene expression control: lessons learned from the vitamin D receptor. Nucleic Acids Res..

[69] Rinaldi L (2022). The glucocorticoid receptor associates with the cohesin loader NIPBL to promote long-range gene regulation. Sci. Adv..

[70] Tan Y (2023). Signal-induced enhancer activation requires Ku70 to read topoisomerase1-DNA covalent complexes. Nat. Struct. Mol. Biol..

[71] Nair SJ (2019). Phase separation of ligand-activated enhancers licenses cooperative chromosomal enhancer assembly. Nat. Struct. Mol. Biol..

[72] Le Dily F (2019). Hormone-control regions mediate steroid receptor-dependent genome organization. Genome Res..

[73] El Khattabi L (2019). A pliable mediator acts as a functional rather than an architectural bridge between promoters and enhancers. Cell.

[74] Soutourina J (2018). Transcription regulation by the mediator complex. Nat. Rev. Mol. Cell Biol..

[75] André KM, Sipos EH, Soutourina J (2021). Mediator roles going beyond transcription. Trends Genet..

[76] Furlong EEM, Levine M (2018). Developmental enhancers and chromosome topology. Science.

[77] Burgess-Beusse B (2002). The insulation of genes from external enhancers and silencing chromatin. Proc. Natl. Acad. Sci. USA.

[78] Lewis AE (2011). Identification of nuclear phosphatidylinositol 4,5-bisphosphate-interacting proteins by neomycin extraction. Mol. Cell Proteom..

[79] Benabdallah NS (2019). Decreased enhancer-promoter proximity accompanying enhancer activation. Mol. Cell.

[80] Williamson I, Lettice LA, Hill RE, Bickmore WA (2016). Shh and ZRS enhancer colocalisation is specific to the zone of polarising activity. Development.

[81] Larsson AJM (2019). Genomic encoding of transcriptional burst kinetics. Nature.

[82] Donovan, B. T. et al. Live-cell imaging reveals the interplay between transcription factors, nucleosomes, and bursting. *EMBO J.* 38. 10.15252/embj.2018100809 (2019). 10.15252/embj.2018100809 PMC6576174 31101674

[83] Senecal A (2014). Transcription factors modulate c-Fos transcriptional bursts. Cell Rep..

[84] Bartman CR (2019). Transcriptional burst initiation and polymerase pause release are key control points of transcriptional regulation. Mol. Cell.

[85] Fukaya T, Lim B, Levine M (2016). Enhancer control of transcriptional bursting. Cell.

[86] Tunnacliffe E, Chubb JR (2020). What is a transcriptional burst?. Trends Genet..

[87] Jin F (2013). A high-resolution map of the three-dimensional chromatin interactome in human cells. Nature.

[88] Panigrahi A, O’Malley BW (2021). Mechanisms of enhancer action: the known and the unknown. Genome Biol..

[89] Panigrahi AK (2018). SRC-3 coactivator governs dynamic estrogen-induced chromatin looping interactions during transcription. Mol. Cell.

[90] Core LJ (2012). Defining the status of RNA polymerase at promoters. Cell Rep..

[91] Baek I, Friedman LJ, Gelles J, Buratowski S (2021). Single-molecule studies reveal branched pathways for activator-dependent assembly of RNA polymerase II pre-initiation complexes. Mol. Cell.

[92] Vernimmen D, De Gobbi M, Sloane-Stanley JA, Wood WG, Higgs DR (2007). Long-range chromosomal interactions regulate the timing of the transition between poised and active gene expression. EMBO J..

[93] Koch F (2011). Transcription initiation platforms and GTF recruitment at tissue-specific enhancers and promoters. Nat. Struct. Mol. Biol..

[94] Kagey MH (2010). Mediator and cohesin connect gene expression and chromatin architecture. Nature.

[95] Sun FH (2021). HPF1 remodels the active site of PARP1 to enable the serine ADP-ribosylation of histones. Nat. Commun..

[96] Adelman K, Lis JT (2012). Promoter-proximal pausing of RNA polymerase II: emerging roles in metazoans. Nat. Rev. Genet..

[97] Schaukowitch K, Kim TK (2014). Emerging epigenetic mechanisms of long non-coding RNAs. Neuroscience.

[98] Chen FX (2017). PAF1 regulation of promoter-proximal pause release via enhancer activation. Science.

[99] Henriques T (2018). Widespread transcriptional pausing and elongation control at enhancers. Genes Dev..

[100] Hnisz D, Shrinivas K, Young RA, Chakraborty AK, Sharp PA (2017). A phase separation model for transcriptional control. Cell.

[101] Sharp PA, Chakraborty AK, Henninger JE, Young RA (2022). RNA in formation and regulation of transcriptional condensates. RNA.

[102] Henninger JE (2021). RNA-mediated feedback control of transcriptional condensates. Cell.

[103] Shrinivas K (2019). Enhancer features that drive formation of transcriptional condensates. Mol. Cell.

[104] Guo YE (2019). Pol II phosphorylation regulates a switch between transcriptional and splicing condensates. Nature.

[105] Sabari, B. R. et al. Coactivator condensation at super-enhancers links phase separation and gene control. *Science* 361. 10.1126/science.aar3958 (2018). 10.1126/science.aar3958 PMC6092193 29930091

[106] Ferrie JJ, Karr JP, Tjian R, Darzacq X (2022). “Structure”-function relationships in eukaryotic transcription factors: the role of intrinsically disordered regions in gene regulation. Mol. Cell.

[107] Ottoz DSM, Berchowitz LE (2020). The role of disorder in RNA binding affinity and specificity. Open Biol..

[108] Bosch J (2019). Effects of blood pressure and lipid lowering on cognition: Results from the HOPE-3 study. Neurology.

[109] Hnisz D, Young RA (2017). New insights into genome structure: genes of a feather stick together. Mol. Cell.

[110] Graham, T. G. W., Ferrie, J. J., Dailey, G. M., Tjian, R. & Darzacq, X. Detecting molecular interactions in live-cell single-molecule imaging with proximity-assisted photoactivation (PAPA). *Elife* 11. 10.7554/eLife.76870 (2022). 10.7554/eLife.76870 PMC9531946 35976226

[111] Abraham BJ (2017). Small genomic insertions form enhancers that misregulate oncogenes. Nat. Commun..

[112] Claringbould A, Zaugg JB (2021). Enhancers in disease: molecular basis and emerging treatment strategies. Trends Mol. Med..

[113] Chen H (2018). A pan-cancer analysis of enhancer expression in nearly 9000 patient samples. Cell.

[114] Deniz Ö (2020). Endogenous retroviruses are a source of enhancers with oncogenic potential in acute myeloid leukaemia. Nat. Commun..

[115] Liu X (2023). Resurrection of endogenous retroviruses during aging reinforces senescence. Cell.

[116] Stephens PJ (2011). Massive genomic rearrangement acquired in a single catastrophic event during cancer development. Cell.

[117] Zhang Y (2020). High-coverage whole-genome analysis of 1220 cancers reveals hundreds of genes deregulated by rearrangement-mediated cis-regulatory alterations. Nat. Commun..

[118] Katainen R (2015). CTCF/cohesin-binding sites are frequently mutated in cancer. Nat. Genet..

[119] Akdemir KC (2020). Disruption of chromatin folding domains by somatic genomic rearrangements in human cancer. Nat. Genet..

[120] Cho SW (2018). Promoter of lncRNA gene PVT1 is a tumor-suppressor DNA boundary element. Cell.

[121] Kimura S (2022). Enhancer retargeting of CDX2 and UBTF::ATXN7L3 define a subtype of high-risk B-progenitor acute lymphoblastic leukemia. Blood.

[122] Shu C (2022). ERR-activated GPR35 promotes immune infiltration level of macrophages in gastric cancer tissues. Cell Death Discov..

[123] Ajima, R., Sakakibara, Y., Sakurai-Yamatani, N., Muraoka, M. & Saga, Y. Formal proof of the requirement of MESP1 and MESP2 in mesoderm specification and their transcriptional control via specific enhancers in mice. *Development* 148. 10.1242/dev.194613 (2021). 10.1242/dev.194613 34679163

[124] Lower KM (2009). Adventitious changes in long-range gene expression caused by polymorphic structural variation and promoter competition. Proc. Natl. Acad. Sci. USA.

[125] Topfer SK (2022). Disrupting the adult globin promoter alleviates promoter competition and reactivates fetal globin gene expression. Blood.

[126] Bateman, J. R. & Johnson, J. E. Altering enhancer-promoter linear distance impacts promoter competition in cis and in trans. *Genetics* 222. 10.1093/genetics/iyac098 (2022). 10.1093/genetics/iyac098 PMC9434180 35748724

[127] Nakamura T (2024). Topologically associating domains define the impact of de novo promoter variants on autism spectrum disorder risk. Cell Genom..

[128] Ong CT, Corces VG (2014). CTCF: an architectural protein bridging genome topology and function. Nat. Rev. Genet..

[129] Zheng H, Xie W (2019). The role of 3D genome organization in development and cell differentiation. Nat. Rev. Mol. Cell Biol..

[130] Dehingia B, Milewska M, Janowski M, Pękowska A (2022). CTCF shapes chromatin structure and gene expression in health and disease. EMBO Rep..

[131] Kemp CJ (2014). CTCF haploinsufficiency destabilizes DNA methylation and predisposes to cancer. Cell Rep..

[132] Konrad EDH (2019). CTCF variants in 39 individuals with a variable neurodevelopmental disorder broaden the mutational and clinical spectrum. Genet Med..

[133] Davis L, Onn I, Elliott E (2018). The emerging roles for the chromatin structure regulators CTCF and cohesin in neurodevelopment and behavior. Cell Mol. Life Sci..

[134] Filippova GN (2002). Tumor-associated zinc finger mutations in the CTCF transcription factor selectively alter tts DNA-binding specificity. Cancer Res..

[135] Bailey CG (2021). Structure-function relationships explain CTCF zinc finger mutation phenotypes in cancer. Cell Mol. Life Sci..

[136] Poulos RC (2016). Functional mutations form at CTCF-cohesin binding sites in melanoma due to uneven nucleotide excision repair across the Motif. Cell Rep..

[137] Kaiser VB, Taylor MS, Semple CA (2016). Mutational biases drive elevated rates of substitution at regulatory sites across cancer types. PLoS Genet..

[138] Guo YA (2018). Mutation hotspots at CTCF binding sites coupled to chromosomal instability in gastrointestinal cancers. Nat. Commun..

[139] Cheng H, Zhang N, Pati D (2020). Cohesin subunit RAD21: from biology to disease. Gene.

[140] Boudaoud I (2017). Connected gene communities underlie transcriptional changes in cornelia de lange syndrome. Genetics.

[141] Weiss FD (2021). Neuronal genes deregulated in Cornelia de Lange Syndrome respond to removal and re-expression of cohesin. Nat. Commun..

[142] Waldman T (2020). Emerging themes in cohesin cancer biology. Nat. Rev. Cancer.

[143] Losada A (2014). Cohesin in cancer: chromosome segregation and beyond. Nat. Rev. Cancer.

[144] Viny AD (2019). Cohesin members Stag1 and Stag2 display distinct roles in chromatin accessibility and topological control of HSC self-renewal and differentiation. Cell Stem Cell.

[145] Wutz, G. et al. ESCO1 and CTCF enable formation of long chromatin loops by protecting cohesin. *Elife* 9. 10.7554/eLife.52091 (2020). 10.7554/eLife.52091 PMC7054000 32065581

[146] Cuadrado A (2019). Specific contributions of cohesin-SA1 and Cohesin-SA2 to TADs and polycomb domains in embryonic stem cells. Cell Rep..

[147] Pang D, Yu S, Yang X (2022). A mini-review of the role of condensin in human nervous system diseases. Front. Mol. Neurosci..

[148] Martin CA (2016). Mutations in genes encoding condensin complex proteins cause microcephaly through decatenation failure at mitosis. Genes Dev..

[149] Golfier, S., Quail, T., Kimura, H. & Brugués, J. Cohesin and condensin extrude DNA loops in a cell cycle-dependent manner. *Elife* 9. 10.7554/eLife.53885 (2020). 10.7554/eLife.53885 PMC7316503 32396063

[150] Cutts EE, Vannini A (2020). Condensin complexes: understanding loop extrusion one conformational change at a time. Biochem. Soc. Trans..

[151] Kakui Y (2020). Fission yeast condensin contributes to interphase chromatin organization and prevents transcription-coupled DNA damage. Genome Biol..

[152] Verheul TCJ, van Hijfte L, Perenthaler E, Barakat TS (2020). The Why of YY1: mechanisms of transcriptional regulation by Yin Yang 1. Front. Cell Dev. Biol..

[153] Dong X (2022). YY1 safeguard multidimensional epigenetic landscape associated with extended pluripotency. Nucleic Acids Res..

[154] Schug J (2005). Promoter features related to tissue specificity as measured by Shannon entropy. Genome Biol..

[155] Khachigian LM (2018). The Yin and Yang of YY1 in tumor growth and suppression. Int. J. Cancer.

[156] Pabian-Jewuła S, Bragiel-Pieczonka A, Rylski M (2022). Ying Yang 1 engagement in brain pathology. J. Neurochem..

[157] Amir RE (1999). Rett syndrome is caused by mutations in X-linked MECP2, encoding methyl-CpG-binding protein 2. Nat. Genet..

[158] Tillotson R, Bird A (2020). The molecular basis of MeCP2 function in the brain. J. Mol. Biol..

[159] Horike S, Cai S, Miyano M, Cheng JF, Kohwi-Shigematsu T (2005). Loss of silent-chromatin looping and impaired imprinting of DLX5 in Rett syndrome. Nat. Genet..

[160] Grubert F (2020). Landscape of cohesin-mediated chromatin loops in the human genome. Nature.

[161] van Arensbergen J, van Steensel B, Bussemaker HJ (2014). In search of the determinants of enhancer-promoter interaction specificity. Trends Cell Biol..

[162] Ahn JH (2021). Phase separation drives aberrant chromatin looping and cancer development. Nature.

[163] Chen M, Liu X, Liu Q, Shi D, Li H (2023). 3D genomics and its applications in precision medicine. Cell Mol. Biol. Lett..

[164] Jia Q, Chen S, Tan Y, Li Y, Tang F (2020). Oncogenic super-enhancer formation in tumorigenesis and its molecular mechanisms. Exp. Mol. Med..

[165] Feehley T, O’Donnell CW, Mendlein J, Karande M, McCauley T (2023). Drugging the epigenome in the age of precision medicine. Clin. Epigenet..

[166] Bauer DE (2013). An erythroid enhancer of BCL11A subject to genetic variation determines fetal hemoglobin level. Science.

[167] Frangoul H (2021). CRISPR-Cas9 gene editing for sickle cell disease and beta-Thalassemia. N. Engl. J. Med..

[168] Erdős G, Pajkos M, Dosztányi Z (2021). IUPred3: prediction of protein disorder enhanced with unambiguous experimental annotation and visualization of evolutionary conservation. Nucleic Acids Res..

[169] Gregor A (2013). De novo mutations in the genome organizer CTCF cause intellectual disability. Am. J. Hum. Genet..

[170] Bastaki F (2017). Identification of a novel CTCF mutation responsible for syndromic intellectual disability - a case report. BMC Med. Genet..

[171] Guo J, Cao B, Xu X, Wu F, Zhu B (2018). Novel CTCF mutations in Chinese patients with ovarian endometriosis. Mol. Med. Rep..

[172] Marshall AD (2017). CTCF genetic alterations in endometrial carcinoma are pro-tumorigenic. Oncogene.

[173] Chatterjee S (2016). Enhancer variants synergistically drive dysfunction of a gene regulatory network in hirschsprung disease. Cell.

[174] Zhang M (2019). Long-range. Proc. Natl. Acad. Sci. USA.

[175] Zhang Z (2021). SNP rs4971059 predisposes to breast carcinogenesis and chemoresistance via TRIM46-mediated HDAC1 degradation. EMBO J..

[176] Gao P (2018). Biology and clinical implications of the 19q13 aggressive prostate cancer susceptibility locus. Cell.

[177] Yu CY (2020). A 16q22.1 variant confers susceptibility to colorectal cancer as a distal regulator of ZFP90. Oncogene.

[178] Giorgio E (2015). A large genomic deletion leads to enhancer adoption by the lamin B1 gene: a second path to autosomal dominant adult-onset demyelinating leukodystrophy (ADLD). Hum. Mol. Genet..

[179] Lupiáñez DG (2015). Disruptions of topological chromatin domains cause pathogenic rewiring of gene-enhancer interactions. Cell.

[180] Lupiáñez DG, Spielmann M, Mundlos S (2016). Breaking TADs: how alterations of chromatin domains result in disease. Trends Genet..

[181] Guo Y (2015). CRISPR inversion of CTCF sites alters genome topology and enhancer/promoter function. Cell.

[182] Flavahan WA (2016). Insulator dysfunction and oncogene activation in IDH mutant gliomas. Nature.

[183] Hnisz D (2016). Activation of proto-oncogenes by disruption of chromosome neighborhoods. Science.

[184] Gröschel S (2014). A single oncogenic enhancer rearrangement causes concomitant EVI1 and GATA2 deregulation in leukemia. Cell.

[185] Northcott PA (2014). Enhancer hijacking activates GFI1 family oncogenes in medulloblastoma. Nature.

[186] Ji X (2016). 3D chromosome regulatory landscape of human pluripotent cells. Cell Stem Cell.

[187] de Bruijn SE (2020). Structural variants create new topological-associated domains and ectopic retinal enhancer-gene contact in dominant retinitis pigmentosa. Am. J. Hum. Genet..

[188] Laugsch M (2019). Modeling the pathological long-range regulatory effects of human structural variation with patient-specific hiPSCs. Cell Stem Cell.

[189] Vicente-García C (2017). Regulatory landscape fusion in rhabdomyosarcoma through interactions between the PAX3 promoter and FOXO1 regulatory elements. Genome Biol..

[190] Chai P (2020). Generation of onco-enhancer enhances chromosomal remodeling and accelerates tumorigenesis. Nucleic Acids Res..

[191] Meinel, J. A. et al. Disruption of the topologically associated domain at Xp21.2 is related to 46,XY gonadal dysgenesis. *J. Med. Genet*. 10.1136/jmg-2022-108635 (2022). 10.1136/jmg-2022-108635 PMC10176412 36227713

[192] Sun JH (2018). Disease-associated short tandem repeats co-localize with chromatin domain boundaries. Cell.

[193] Valton AL, Dekker J (2016). TAD disruption as oncogenic driver. Curr. Opin. Genet. Dev..

[194] Kaiser, V. B. & Semple, C. A. When TADs go bad: chromatin structure and nuclear organisation in human disease. *F1000Res* 6. 10.12688/f1000research.10792.1 (2017). 10.12688/f1000research.10792.1 PMC5373421 28408976

[195] Anania C, Lupiáñez DG (2020). Order and disorder: abnormal 3D chromatin organization in human disease. Brief. Funct. Genom..

[196] Tena JJ, Santos-Pereira JM (2021). Topologically associating domains and regulatory landscapes in development, evolution and disease. Front. Cell Dev. Biol..

[197] Chen F (2019). Three additional de novo CTCF mutations in Chinese patients help to define an emerging neurodevelopmental disorder. Am. J. Med. Genet C. Semin. Med. Genet..

[198] Oh S, Oh C, Yoo KH (2017). Functional roles of CTCF in breast cancer. BMB Rep..

[199] Price E (2023). An updated catalog of CTCF variants associated with neurodevelopmental disorder phenotypes. Front. Mol. Neurosci..

[200] Zhang BN (2019). A cohesin subunit variant identified from a peripheral sclerocornea pedigree. Dis. Markers.

[201] Bonora E (2015). Mutations in RAD21 disrupt regulation of APOB in patients with chronic intestinal pseudo-obstruction. Gastroenterology.

[202] Shah RR, Bird AP (2017). MeCP2 mutations: progress towards understanding and treating Rett syndrome. Genome Med..

[203] Gabriele M (2017). YY1 haploinsufficiency causes an intellectual disability syndrome featuring transcriptional and chromatin dysfunction. Am. J. Hum. Genet..

